# Application of a Negative Intrauterine Pressure Suction Device for Prophylactic Management of Atonic Postpartum Hemorrhage: A Quality Improvement Study

**DOI:** 10.7759/cureus.42631

**Published:** 2023-07-28

**Authors:** Jagadish C Sharma, Pragathi Kollabathula, Sonam Jindal, Anupma Anupma, Avir Sarkar, Saroj Jaggarwal, Ramesh Chandra, Lokesh Parashar

**Affiliations:** 1 Obstetrics and Gynecology, Employees' State Insurance Corporation (ESIC) Medical College and Hospital, Faridabad, Faridabad, IND; 2 Obstetrics and Gynecology, Employees' State Insurance Corporation (ESIC) Medical college and Hospital, Faridabad, Faridabad, IND; 3 Community Medicine, Employees' State Insurance Corporation (ESIC) Medical College and Hospital, Faridabad, Faridabad, IND

**Keywords:** suction cannula, negative intrauterine pressure suction device, uterotonics, active management of third stage of labor, postpartum hemorrhage

## Abstract

Background: Postpartum hemorrhage (PPH) is the leading cause of maternal mortality worldwide. PPH-preventing interventions need to be prioritized and can be integrated with conventional methods of PPH prevention. The introduction of negative intrauterine pressure using a suction cannula can be one of the cheapest modalities to decrease PPH secondary to uterine atonicity. This method has brought a renaissance to practical obstetrics in low-middle income countries (LMIC), where the cost and availability of uterotonics are major health issues.

Methods: It was a prospective quality improvement (QI) study conducted in the labor and delivery wards of a tertiary care medical institute and teaching center over the duration of one year. We aimed to assess the decrease in the incidence of atonic PPH with a negative intrauterine pressure suction device (NIPSD) integrated with active management of the third stage of labor (AMTSL) in the prevention of atonic PPH following normal vaginal delivery in low-risk antenatal women. In the initial six months, routine AMTSL was instituted for all consenting women (group 1). In the next six months, NIPSD was integrated with AMTSL (group 2). Data pertaining to the amount of blood loss, the incidence of primary PPH, uterine tone, fall in hemoglobin and hematocrit levels post-delivery, need for blood transfusion, and doctor and patient satisfaction were tabulated for all patients.

Results: A total of 1324 consenting women were eligible for enrollment during the study time frame. In the initial six months (baseline period, group 1), 715 participants were subjected to routine AMTSL in the third stage of labor. During the intervention phase (group 2), 609 parturient women were recruited. There was no significant difference in baseline parameters between the two groups. With the introduction of NIPSD to routine AMTSL, there was a significant decrease in the average volume of blood loss during vaginal delivery (group 1 = 389.45+65.42 ml, group 2 = 216.66+34.27 ml; p-value = 0.012). The incidence of atonic PPH was reduced by more than 75% (group 1 = 13 women, group 2 = 3 women; p-value = 0.001) after the introduction of NIPSD complementing routine AMTSL. The introduction of NIPSD has also been instrumental in reducing the cost burden on patient and hospital expenditures. The net benefit of its introduction resulted in a reduction of the overall cost burden of blood transfusions by around 70%.

Conclusion: PPH is a public health problem, and measures to reduce PPH must be implemented to decrease this health burden. In countries with low resources, complementing routine AMTSL with NIPSD can be instrumental in decreasing the incidence of PPH. Considering its cost-effectiveness and reusability, LMIC can adopt NIPSD as a routine measure in all vaginal deliveries.

## Introduction

Postpartum hemorrhage (PPH) is the most dreadful obstetric emergency and is a leading cause of maternal death in low-income countries, with an incidence as high as one in 45 among low-resource settings [1]. Every year, 14 million women around the world suffer from PPH [1]. It is estimated that worldwide, 1,40,000 women die of PPH annually. It accounts for almost one maternal mortality every four minutes! PPH contributes to nearly one-third of all maternal deaths worldwide [2].

The maternal mortality rate (MMR) in Indian women stands at a figure of 97 per one lakh live births [1,2]. However, PPH still remains the leading cause of maternal mortality and morbidity, especially in low-middle-income countries (LMIC). The major contributing factors to PPH are anemia, multiparity, and delay in seeking antenatal care, leading to undiagnosed high-risk conditions. The reported incidence of PPH in India is 2-4% after vaginal delivery and 6% after cesarean section, with uterine atony being the most common cause (50%) [3].

Postpartum hemorrhage is defined as blood loss from the genital tract exceeding 500 ml within 24 hours of vaginal delivery and 1000 ml or more during a cesarean section. The American College of Obstetrics and Gynecology (ACOG) defines PPH as the amount of blood loss that decreases the hematocrit by 10% or any amount of blood loss that necessitates a blood transfusion. For clinical purposes, any amount of blood loss that has the potential to produce hemodynamic instability should be considered PPH [4]. PPH-preventing interventions need to be prioritized and can be integrated with conventional methods of PPH prevention. The introduction of negative intrauterine pressure using a suction cannula can be one of the cheapest modalities to decrease PPH secondary to uterine atonicity. This negative pressure acts by sucking on the inner surface of the uterus, thereby mechanically closing all the sinusoids in the endometrium. This method has brought a renaissance to practical obstetrics in the LMIC, where the cost and availability of uterotonics are major health issues.

## Materials and methods

It was a prospective quality improvement (QI) study conducted in the labor and delivery wards of ESIC Medical College and Hospital for a duration of one year. Written informed consent was obtained from all participants. All procedures in the study involving human patients were performed in accordance with the ethical standards of the Institutional Ethics Committee of ESIC Medical College and Hospital (approval number 134 X/11/13/2022-IEC/79) and with the 1964 Helsinki Declaration and its later amendments or comparable ethical standards. Consecutive consenting women undergoing normal vaginal delivery in this tertiary care teaching hospital during the study time frame were recruited into the trial. We aimed to assess the decrease in the incidence of atonic PPH with a negative intrauterine pressure suction device (NIPSD) integrated with active management of the third stage of labor (AMTSL) in the prevention of atonic PPH following normal vaginal delivery in low-risk antenatal women. The objectives were to measure the blood loss after using NIPSD along with AMTSL as compared to the use of AMTSL alone and to assess the tone of the uterus before and after the application of NIPSD. All antenatally consenting women (nulliparous and multiparous) with both spontaneous and induced labor were recruited during active labor. Exclusion criteria consisted of women undergoing cesarean delivery, women with a previously scarred uterus who were at risk of scar dehiscence during the trial of labor, participants sustaining genital tract trauma (cervical tear or obstetric anal sphincter injuries) during delivery, women with diagnosed uterine anomalies or known cases of inherited and acquired coagulopathies, and patients with evidence of clinical or biochemical chorioamnionitis.

After a thorough history, general physical examination, and obstetrical examination, all antenatal mothers admitted to the labor room for normal vaginal delivery were recruited during the active stage of labor (5 cm of cervical dilatation and beyond). For the initial six months (baseline phase), women undergoing vaginal delivery and consenting to participate in the QI project were subjected to routine AMTSL (administration of 10 IU of oxytocin through the intravenous route slowly within one minute of delivery, placental delivery by controlled cord traction [CCT], and assessment of uterine tone) in the third stage of labor as per the hospital protocol (group 1). The incidence of primary PPH and uterine atonicity was assessed in all recruited women. For the next six months (intervention phase, group 2), NIPSD along with AMTSL was provided to all participants (application of an intrauterine negative pressure suction cannula for 30 minutes with a suction pressure of 650 mm of mercury after ruling out cervical and vaginal tears in the fourth stage of labor, in addition to AMTSL). No separate inclusion criteria were used for assigning groups to the participants. The incidence of atonic PPH was also noted. Blood for hemoglobin and hematocrit was sent at the onset of active labor and again 24 hours after delivery for post-delivery assessment. Data pertaining to the amount of blood loss, the incidence of primary PPH, uterine tone, fall in hemoglobin and hematocrit levels post-delivery, need for blood transfusion, and doctor and patient satisfaction were tabulated for all patients. Patient and doctor satisfaction were rated according to the five-point semi-quantitative Likert scale (ranging from zero to a maximum of five points, with zero signifying the worst satisfaction and five signifying the maximum satisfaction with the said treatment modality). A comparison was made in the above parameters between the two groups of participants (AMTSL alone versus NIPSD with AMTSL), and bivariate analysis was done to calculate the p-value for each parameter.

All the collected data were tabulated in a Microsoft Excel worksheet (Microsoft, Redmond, WA, USA), and statistical analysis was performed by SPSS software 23.0 (IBM Corp., Armonk, NY, USA). Rates and proportions were calculated for categorical variables. A bivariate analysis using an independent Student t-test and Fisher’s exact was used to compare the demographic features between women with AMTSL alone and NIPSD with AMTSL. Continuous variables were presented as mean + SD. A p-value of <0.05 was considered statistically significant.

## Results

A total of 1324 consenting women were eligible for enrollment during the study time frame. In the initial six months (baseline period, group 1), 715 participants were subjected to routine AMTSL in the third stage of labor. During the intervention phase (group 2), 609 parturient women were recruited. The demographic features of the study population are tabulated in Table 1. The mean age of the participants in both groups was comparable (p-value = 0.784). The majority of the recruited women were primigravidae (group 1 = 57.06% and group 2 = 62.56%; p-value = 0.656), educated from 6th to 12th standard (group 1 = 79.72% and group 2 = 77.67%; p-value = 0.562), and belonged to lower socioeconomic classes (lower and upper lower combined) according to the Modified Kuppuswamy scale (group 1 = 55.52% and group 2 = 60.26%; p-value = 0.165). Most of the participants practised Hinduism as their religion (group 1 = 92.02% and group 2 = 90.80%; p-value = 0.604). There was no statistical difference in the number of recruited women with anemia between the two groups (p-value = 0.375). Also, history pertaining to PPH in previous pregnancies was elicited from all multigravida patients. There was no significant difference between the women of both groups (p-value = 0.712).

**Table 1:** Comparison of demographic features of the population under study AMTSL: active management of third stage of labor; NIPSD: negative intrauterine pressure suction device; PPH: postpartum hemorrhage)

Assessments	Parameters	Group 1 (AMTSL alone) [n=715]	Group 2 (NIPSD + AMTSL) [n=609]	p-value
Age (in years)		25.01+3.42	25.72+2.52	0.784
Parity	Primigravida	408(57.06%)	381(62.56%)	0.656
	Multigravida	307(42.94%)	228(37.44%)	
Education Status	Upto 5^th^ standard	63(8.81%)	61(10.09%)	0.562
	6^th^ to 12^th^ standard	570(79.72%)	473(77.67%)	
	Graduate and Post-graduate	82(11.46%)	75(12.32%)	
Socio-economic status	Lower	397(55.52%)	367(60.26%)	0.165
	Middle	257(35.94%)	186(30.54%)	
	Upper	61(8.53%)	56(9.19%)	
Religion	Hinduism	658(92.02%)	553(90.80%)	0.604
	Islam	51(7.13%)	54(8.87%)	
	Sikhism	6(0.84%)	2(0.32%)	
History of PPH in previous pregnancy (among multigravida)		14(4.56%)	9(3.94%)	0.712
Anemia in current pregnancy		493(68.95%)	435(71.42%)	0.375

Table 2 compares the study parameters in groups 1 and 2. With the introduction of NIPSD to routine AMTSL, there was a significant decrease in the average volume of blood loss during vaginal delivery (group 1 = 389.45 + 65.42 ml, group 2 = 216.66 + 34.27 ml; p-value = 0.012). The incidence of atonic PPH was reduced by more than 75% (group 1 = 13 women, group 2 = 3 women; p-value = 0.001) after the introduction of NIPSD complementing routine AMTSL. Although drops in hemoglobin and hematocrit were not statistically significant in both groups (p-values of 0.125 and 0.330, respectively), NIPSD was associated with a minimal decrease in both parameters compared to AMTSL alone. It was worth noting that the need for blood and blood product transfusion decreased to less than one-third of the previous requirement when a suction cannula was introduced in the labor wards (p-value = 0.001). Of the 12 transfusions that were done after the introduction of NIPSD, six were random donor platelet transfusions in a patient with aplastic anemia during delivery, two were packed red cell transfusions in an unbooked patient with severe anemia presenting in labor, and four were fresh frozen plasma transfused to a patient with autoimmune hepatitis and a deranged coagulogram profile just prior to delivery. No other patient required blood transfusion in group 2 as compared to group 1, where a total of 38 units of blood products were transfused over a six-month duration (Table 2).

**Table 2:** Comparison of study parameters in both the groups of participants AMTSL: active management of third stage of labor; NIPSD: negative intrauterine pressure suction device; PPH: postpartum hemorrhage

Parameters	Group 1 (AMTSL alone) [n=715]	Group 2 (NIPSD + AMTSL) [n=609]	P-value
Blood loss (in milliliters)	389.45+65.42	216.66+34.27	0.012
Atonic PPH	13(1.81%)	3(0.49%)	0.001
Fall in hemoglobin (in grams/deciliters)	1.69+0.28	1.45+0.33	0.125
Fall in hematocrit (in percentage)	4.95+0.77	4.47+0.89	0.330
Need for transfusion of blood and blood products	38(5.31%)	12(1.97%)	0.001
Net expenditure on blood and blood products (in rupees)	40,000–53,200	9800–14,600	-
Mean patient satisfaction (Likert scale)	5	5	-
Mean doctor satisfaction (Likert scale)	4	5	-

The introduction of NIPSD has also been instrumental in reducing the cost burden on patients and hospital expenditures in our study. No previous study has been conducted to identify and assess the available evidence related to the cost-effectiveness of uterine NIPSD. Considering the clinical scenario in which the study has been conducted, procurement of one unit of packed red cell concentrate costs Rs. 1500 to Rs. 2500, while each unit of random donor platelets and fresh frozen plasma costs Rs. 500 to Rs. 800, respectively. The additional burden of expenditure due to blood grouping and cross-matching cannot be forgotten. Calculating the cost burden incurred upon the hospital system due to blood and blood product transfusion during the study time frame, it was estimated that the net expenditure reduced to less than one-fourth after the introduction of NIPSD (group 1 = 40,000 to 53,200 rupees, group 2 = 9800 to 14,600 rupees). The metallic NIP suction cannula is reusable and is readily available in the market at an affordable cost (around 1500 rupees). Considering the moderate delivery load of around 95 to 118 vaginal deliveries per month, two reusable metallic NIP suction cannulae were sufficient for this setup. If the cost of purchasing a suction cannula were added (2 × 1500 = 3000 rupees), even then the net benefit of its introduction would result in a reduction of the overall cost burden by around 70%.

Although the introduction of NIPSD to routine AMTSL did not bring about a change in patient satisfaction according to the Likert scale, obstetricians reported that they found it better to use the suction cannula. A reduction in the incidence of atonic PPH resulted in lesser anxiety among the treating obstetricians (Table 2).

## Discussion

According to the Millennium Development Goal, maternal mortality reduction can be achieved if only PPH management is prioritized [5]. WHO has emphasized that maternal morbidity and mortality due to PPH can be prevented with prompt recognition and timely management [6-10]. AMTSL has been made a part of safe delivery practices in order to prevent PPH [5,6]. It has been widely practiced throughout the world, leading to a decrease in the incidence of PPH over the past few decades. Despite a decrease in incidence, PPH still remains the major cause of maternal mortality in developing countries like India. Hence, there is a need for additional practices and interventions in order to decrease the disease burden and improve the health of women. PPH-preventing interventions such as negative suction devices assist the natural contractility and retractility of uterine musculature and aid in preventing atonicity of the uterus and blood loss when integrated with conventional AMTSL. Such additional measures are instrumental in decreasing blood loss in the third stage of labor and preventing postpartum hemorrhage [11-14].

Tondge and Burande retrospectively studied 168 patients suffering from PPH by record analysis with respect to maternal age, parity, socio-demographic and etiological profile, and maternal consequences. They showed that uterine atonicity was the most common cause of PPH (69%) leading to 20% of maternal deaths due to hemorrhage. The study concluded that proper anticipation and skilled management, along with timely referral of PPH cases, lead to a significant reduction in maternal morbidity and mortality [12]. Shanthi and Chitra conducted a randomized trial on 300 antenatal mothers, divided into three groups of 100 each, in which one group was given AMTSL, the second group was given AMTSL without uterine massage, and in the third group, AMTSL with the application of a suction cannula was applied. It was found that blood loss was significantly reduced in the AMTSL with cannula group (223+99.2) when compared to mean blood loss in the routine AMTSL-only group (299.0+103.0) and AMTSL without uterine massage group (280.8+119.5), concluding that vacuum retraction cannula is helpful in maintaining the uterine physiology of normal contractility and retractility with a statistically significant level of difference (p≤0.01) [13].

Thangaraju et al. conducted an RCT for the comparison of high-dose and low-dose continuous infusions of oxytocin in the prevention and management of PPH. Outcomes were measured by the additional need for uterotonic medications to control hemorrhage and the assessment of vital volumes. This study concluded that lower doses are preferred over higher doses; the incidence of uterine atony in the low-dose group was 0.7% and in the high-dose group was 5.1%. It indicates that higher doses cause uterine atony because of receptor desensitization, resulting in ineffective action on the uterine myometrium, and concludes that there is no difference in the regular dose (10 U) and high-dose groups as low doses are better tolerated and have similar efficacy [14]. WHO recommendations for the prevention and treatment of PPH stated that oxytocin (10 U, IV/IM) is the recommended uterotonic of choice. Delayed cord clamping (after one to three minutes) and postpartum uterine tone assessment for early identification of uterine atony are strongly recommended. Controlled cord traction and sustained uterine massage are weak recommendations for the prevention of PPH [5].

The World Health Organization (March 2022) published recommendations on intrapartum care for a positive childbirth experience, which recommended oxytocin (10 IU, IM/IV) as the preferred uterotonic drug for the prevention of postpartum hemorrhage. Other injectable uterotonics (ergometrine/methyl ergometrine) and oral misoprostol (600 µg) can be used when oxytocin is not available. Delayed cord clamping (not earlier than one minute after birth) and controlled cord traction by skilled birth attendants must be done. However, sustained uterine massage is not recommended as an intervention to prevent PPH in women who have received prophylactic oxytocin [6]. Samarthram et al. conducted a case-control study including 16 normally delivered patients (NVD) and 4 caesarean (LSCS) delivered patients who developed atonic PPH and did not respond well to the routine use of injections of oxytocin, methergine, or carboprost. A cannula applied for 10 minutes every hour for three hours by creating a negative pressure of 650 mmHg resulted in complete cessation of bleeding associated with contraction and firm retraction of the uterus within four minutes after the initiation of the procedure, and blood collected in the suction bottle ranged from 150 ml to 250 ml [15].

Panicker et al. conducted a prospective interventional study on 40 women who had NVD and 15 who had LSCS and concluded that negative suction resulted in the aspiration of all the blood collected in the uterine cavity. The quantity of blood sucked varied from 50 to 300 ml. When the collected blood was completely sucked out, the bleeding ceased. The suction was maintained for 30 minutes, which resulted in the contraction of the uterus. In all cases, the bleeding stopped after negative intra-uterine suction pressure, except in five patients who had fresh bleeding even after connecting suction; these were found to have vaginal tears and were sutured. In two patients, the cannula got blocked by blood clots, and replacing the cannula immediately controlled the bleeding [16]. Damor et al. conducted an observational study on 120 patients (74 NVD, 25 operative vaginal delivery, and 21 LSCS) and identified that in 50% of women, bleeding was stopped within three minutes, and in 31.7%, bleeding was stopped within 3-4 minutes. The blood collected in bottles ranged from 100 to 150 ml, concluding that the application of the SR cannula as a novel technique in high-risk women with atonic PPH averts catastrophic bleeding [17].

Meena et al. conducted a prospective observational study on 20 NVD women and 5 LSCS women who developed atonic PPH. In 40% of cases, the bleeding stopped within 3 to 3.9 minutes of application of a suction retraction (SR) cannula for 10 minutes every hour for three hours with a negative pressure of 650 mmHg, and in 24% of cases, the bleeding stopped within 2.1 to 2.9 minutes, and in another 24% of cases, the bleeding stopped within two minutes after application of the cannula. The majority of cases, 23 (92%), survived following the use of the SR cannula [18]. Sowjanya et al. conducted a prospective interventional study on 100 women with different risk factors for PPH, in which SR cannula was used prophylactically on 50 women, and the mean blood loss after the use of SR cannula in study group A was 187.6+83.11; p = 0.00114, whereas in control group B it was 378+157.18; p = 0.00114, and the mean difference of hemoglobin level before and after delivery in the study group is 0.29 (p = 0.125) and in the control group is 0.03 (p = 0.87), indicating that the majority of blood loss was found in the control group. This study concluded that the majority of cases of PPH can be predicted based on risk factors recognized antenatally; hence, prophylactic application of a vacuum retraction cannula intrapartum in identified high-risk cases prevents catastrophic bleeding [19].

The current study has also shown the benefit of introducing NIPSD to routine AMTSL in reducing the incidence of primary atonic PPH and the need for blood transfusions in parturient women. The major advantage is the minimal expenditure associated with a suction cannula. No other previous study has assessed the reduction of expenses due to the introduction of NIPSD in a hospital setting (with the decrease in expenses due to uterotonics). NIP cannulas come in different shapes and sizes and are readily available in the market. The metallic reusable ones can be used any number of times after thorough washing and autoclaving. Considering the true scenario in LMIC, where purchasing uterotonics and the cost burden of storage and refrigeration are major practical concerns, this NIPSD can be a great savior in the lives of many.

This study had a few limitations as well. It was a single-center, non-randomized study. The study was approved for a period of one year. Only vaginal deliveries were included in this trial. PPH during cesarean deliveries was not addressed in the index study. The use of other uterotonics in difficult situations (like severe PPH) would have been a confounding factor in the study. However, risking a life for the sake of a study would not have obtained ethical approval. Large multicentric data and systematic reviews need to be conducted before formulating any guidelines.

## Conclusions

PPH is a public health problem, and measures to reduce PPH must be implemented to decrease this health burden. In countries with low resources, complementing routine AMTSL with NIPSD can be instrumental in decreasing the incidence of PPH. This quality improvement initiative has demonstrated that NIPSD is an effective method of preventing atonic PPH. However, stress must be given on training the ground staff for better awareness and acceptance of this method. LMIC can adopt NIPSD as a routine prophylactic measure for all vaginal deliveries. However, large, long-term, multicentric randomized controlled trials with a huge sample size and diverse ethnic groups are needed before drawing a solid conclusion.
